# Degeneration dependent changes in human knee cartilage mechanical properties revealed by synchrotron tomography based finite element modeling

**DOI:** 10.1016/j.ocarto.2026.100825

**Published:** 2026-05-27

**Authors:** Viktor Jönsson, Lorenzo Grassi, Anna Gustafsson, Maria Pierantoni, Hector Dejea, Amanda Sjögren, Christian M. Schlepütz, Martin Englund, Hanna Isaksson

**Affiliations:** a Department of Biomedical Engineering, Lund University, Lund, Sweden; b MAX IV Laboratory, Lund University, Lund, Sweden; c Clinical Sciences Lund Orthopedics, Lund University, Lund, Sweden; d Swiss Light Source, Paul Scherrer Institute, Villigen, Switzerland

**Keywords:** Articular cartilage, Osteoarthritis, OARSI grade, Stress-relaxation, Fibril-reinforced poroelastic

## Abstract

**Objective:**

Knee osteoarthritis (OA) is a chronic joint disease associated with pain and reduced function. The mechanisms underlying OA and how mechanical properties change with the disease are not fully understood, partly due to large variability during mechanical testing of cartilage. We characterized the mechanical properties of human femoral cartilage with varying levels of degeneration using a tissue constitution specific fibril-reinforced poroelastic (FRPE) material model.

**Method:**

We created sample-specific finite element (FE) models based on synchrotron-based x-ray tomography to reduce the variability caused by sample geometry. For comparison, idealized FE models were also created without tomography data. Cartilage samples (n = 15) were mechanically tested in compressive stress relaxation (two steps of 15% strain) with in-situ x-ray tomography. Adjacent tissue samples were histopathologically graded (OARSI). The FRPE parameters were optimized to minimize differences between experimental and simulated stress relaxation forces. Identified material parameters were analyzed with linear regression, with OARSI grade as independent variable.

**Results:**

We identified reductions in both collagen (-2x) and non-fibrillar matrix stiffness (-3x) accompanied by increased permeability (+5.5x) when comparing tissue with OARSI grade 1 and 5 in tomography-based FE models. At OARSI = 1, in idealized and tomography-based models, the collagen stiffness, non-fibrillar matrix stiffness and permeability differed 20, 110 and 55% respectively. Only collagen and non-fibrillar matrix stiffness were associated with OARSI grade in idealized models.

**Conclusion:**

Segmentation-based models were better at detecting degeneration-related mechanical changes than idealized models. The identified parameter changes match known OA tissue developments and can be used in predictive FE knee joint models.

## 1 Introduction

Knee osteoarthritis (OA) is a common painful joint disease that affects millions of people each year [1]. There is currently no cure available and the number of patients is expected to increase due to an ageing population and an increased prevalence of obesity [1,2]. The disease affects the whole joint with structural degeneration occurring in the articular cartilage, meniscus, ligaments, and subchondral bone [[3], [4], [5]]. Healthy cartilage has exceptional mechanical properties, functioning as a smooth and shock absorbing thin tissue between the nonconforming bones in the joint. As OA progresses, these functions degrade [6]. The mechanisms behind the structural degeneration of articular cartilage during OA are not fully known. Relating structural degeneration to mechanical properties could be one way to better understand the mechanisms at play.

Articular cartilage is mainly composed of water, proteoglycans and collagen type 2 fibrils. The proteoglycans contain fixed negative charges, which results in an osmotic swelling pressure [7,8]. Collagen fibrils reinforce the tissue, constraining the swelling and increasing the fluid pressure [9]. Further, the fibrils are arranged in an arcade pattern and are perpendicular to the subchondral bone–cartilage interface in the deep zone. In the middle zone, they transition toward an orientation parallel to the surface in the superficial zone [10]. Cartilage's low permeability causes the fluid pressure to rise during compressive transient loading, increasing the loadbearing capacity. Cartilage degeneration due to OA leads to alterations in the tissue structure and composition. The proteoglycan content decreases, the collagen network alignment is altered and the collagen density is reduced [[11], [12], [13], [14], [15]]. This causes the tissue to swell with an increased fluid fraction and decreased solid content, increasing permeability while the dynamic and equilibrium stiffness decreases [6,13,16]. These changes reduce the loadbearing capacity of cartilage and impair its function in the knee joint.

Articular cartilage's mechanical properties have been studied using human tissue [[17], [18], [19]] at different stages of degeneration. The mechanical properties have been determined directly from mechanical test data [16,18,20] or by fitting advanced material models using the finite element (FE) method [19,[21], [22], [23]]. Many of these studies assume that all the samples have an ideal shape. To our knowledge, only Robinson et al. created FE models with sample-specific geometries using photo tomography [22]. Based on in-situ synchrotron-radiation-based phase-contrast enhanced micro-tomography (SR-PhC-μCT) of bovine articular cartilage samples [24], we recently showed that the results of unconfined compression are greatly affected by small differences in sample shape [25]. We found that samples with an average top surface inclination (4°) from the assumed flat surface caused a 15% reduction in reaction forces. Adding variability to the mechanical testing and making it more challenging to determine the mechanical properties. By accounting for variability in sample shape the inter-sample variability in mechanical properties can be reduced.

The aim of this study was to determine the mechanical properties of human femoral knee cartilage at different stages of degeneration. This was achieved using segmented, sample-specific FE models based on in-situ SR-PhC-μCT experimental data from human samples under compression. We used a fibril-reinforced poroelastic (FRPE) [26] material model. For comparison, the mechanical properties were also identified using idealized cylindrical FE models. Cartilage degradation was based on histopathological grading using *Osteoarthritis Research Society International* (OARSI) grades [27]. Finally, the mechanical properties were determined directly from the experimental mechanical data and compared with the fibril-reinforced poroelastic properties obtained from material optimization for the segmented models.

## 2 Method

### 2.1 Experimental data

**Fig. 1:**
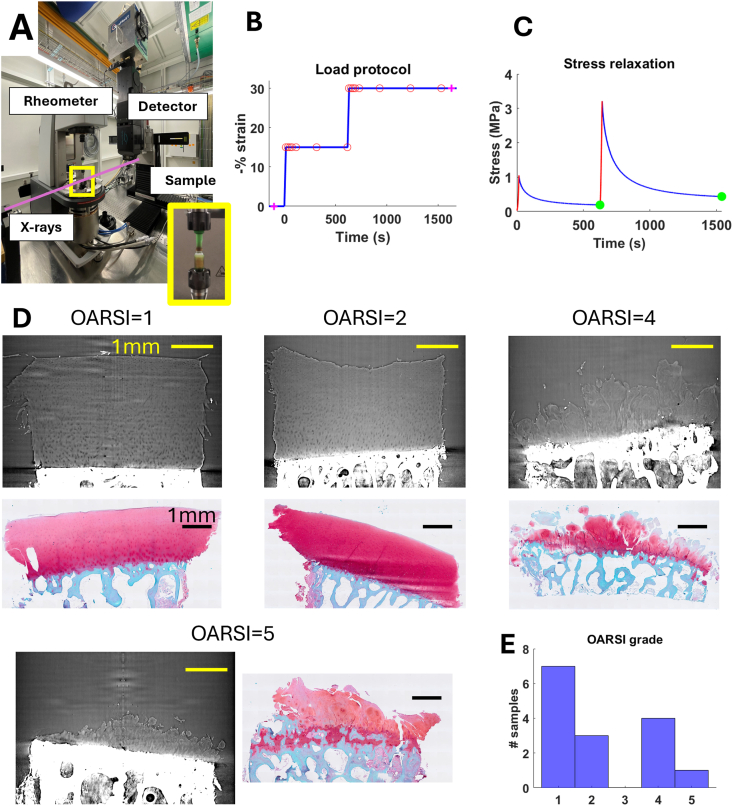
A) Experimental setup with rheometer at beamline with close-up of a mounted sample. B) Loading protocol with image acquisition timepoints marked (magenta line = static images taken before and after loading, red circle = dynamic images taken during loading). C) Typical stress relaxation with loading ramps marked in red used for calculating the compressive modulus, stress at equilibrium marked in green. D) Example SR-PhC-μCT static image longitudinal slices before loading with corresponding histology. E) OARSI grading distribution for all samples without subgrading.

Cylindrical osteochondral plugs (n = 21, diameter 4 mm) were harvested from the medial femoral condyle of 21 male deceased donors (age 20–85 years). Samples were obtained from the MENIX biobank (Skåne University Hospital, Sweden; ethical approvals Dnr 2015–39, 2016-865, and 2019-00323). None of the donors had a clinical diagnosis of arthritis. The samples were tested in compression submerged in PBS, using a rheometer (Anton Paar MCR 702e MultiDrive) while being imaged using SR-PhC-μCT at the TOMCAT beamline, PSI (Swiss Light Source, Villigen, Switzerland) [28] (Fig. 1A). The load cell in the rheometer was limited to 40 N in compression. The 3D-printed sample holders had an inner diameter of 5.5 mm and the indenter diameter was 5 mm. The tomographs had an isotropic voxel size of 2.75 μm and a field of view of 5.54 x 3.85 mm^2^. Static images (scan acquisition time = 40 s) were acquired before and after loading the samples, while dynamic images (scan acquisition time = 5 s) were collected at different relaxation times during stress-relaxation loading (Fig. 1B). We recently presented the proof of concept of the experimental setup and approach for bovine samples, and the reader is referred there for more details [24].

The loading protocol was as follows: A preload of 2 N was applied over 15 s to ensure proper contact between sample and test device, followed by 300 s of relaxation for the sample to equilibrate with the indenter fixed. Then, two displacement steps (15 and 30% compressive strain, load rate 1%/s) were applied followed by 600 and 900 s of relaxation, respectively. The loading protocol with image acquisitions can be seen in Fig. 1B. The applied displacement was determined based on the average height of the cartilage as measured from the x-ray projection images after pre-load.

The elastic equilibrium modulus $E_{eq}$ was determined directly from the stress relaxation data as the slope of the line crossing the stress-strain points at equilibrium for the two stress relaxations (Fig. 1C, green points). The compressive modulus [29] was calculated as the slope of the stress-strain data recorded during the two loading ramps (Fig. 1C, red lines). An example is shown in Supplementary Fig. S1. The total relaxation time was measured as the time it took for each stress relaxation to reach half of the equilibrium force.

### 2.2 Histology

Histology samples were taken adjacent to those used for mechanical testing from the same 16 mm donor osteochondral plug, an example is available in Supplementary Fig. S2. Details for the histology are available in [30] and are only briefly summarized here. First, samples were fixed in 4% formalin and decalcified in EDTA. After, samples were dehydrated in a series of alcohol solutions, cleared with xylene and embedded in paraffin. Five μm thick sections were cut and stained with Weigert's iron hematoxylin and Safranin-O & Fast green (W-SafO-FG). The grading was done in accordance with the OARSI histopathological grading system [27] on pseudonymized images, with consensus grading conducted by two observers [30].

The distribution of the OARSI grades for all samples in this study is presented in Fig. 1E. A visual comparison between the histology and the unloaded SR-PhC-μCT images available in Fig. 1D for samples exhibiting mild to severe degeneration (OARSI = 1–5).

## 3 Sample exclusion

Out of the 21 tested samples, 6 were excluded from analysis. The reasons were overload of the range of the load cell (N = 1), cracks in the sample (probably from sample preparation) that were visible in the unloaded SR-PhC-μCT images (N = 3), and large discrepancy in level of degradation between the two adjacently harvested samples used for synchrotron and histology images (N = 2). More information about the excluded samples is available in Supplementary Fig. S3–4.

## 4 FE modelling

### 4.1 Material model

Cartilage was modelled as a Fibril-Reinforced Poro-hyperElastic (FRPE) [26] material with a solid and an incompressible fluid phase [31]. Fluid flow *q* was assumed to follow Darcy's law [32] (equation (1)). The permeability *k* was assumed to be strain dependent [33] with the initial permeability *k*_*0*_, current and initial void ratio *e* and *e*_*0*_ and exponent *M* (equation (2)) [34]. In the solid phase, the non-fibrillar matrix was represented with a compressible neo-Hookean material given by the non-fibrillar solid stress $\sigma_{s,nf}$ (equation (3)) [35] with the bulk and shear modulus *K* and *G* determined by the non-fibrillar Poisson's ratio $\nu_{nf}$ and Young's modulus $E_{nf}$. The fibril stress $\sigma_{s,nf}$ was given by equation (4) [26] with *E*_*0*_ being the fibril stiffness, and where fibrils only resist tension (fibril strain $\varepsilon_{f}>0$). The total stress ***σ*_*t*_** is the sum of the stresses (equation (5)).

(1) q=−k∇p

(2) $$k=k_{0}{\left(\frac{1+e}{1+e_{0}}\right)}^{M}$$

(3) σs,nf=12KJ−1JI+GJF·FT−J23I,whereK=Enf31−2νnf,andG=Enf21+νnf

(4) $$\sigma_{s,f}=E_{0}\varepsilon_{f},\;\varepsilon_{f}>0$$

(5) $$\sigma_{t}=\sigma_{s,nf}+\sigma_{s,f}-pI$$

The depth-dependent tissue composition and structure was assumed to be the same for all samples irrespective of OARSI grade [21]. The water fraction was set to $0.8-0.15z^{*}$ [36,37] where $z^{*}$ is the normalized sample depth. The collagen density was set to $1.4{\left(z^{*}\right)}^{2}-1.1z^{*}+0.59$ [38,39] and was assumed to have an arcade-like structure [10,26]. The cartilage material model was implemented in Abaqus v2021 (Dassault Systemes) as a user defined material.

### 4.2 Tomography model – simulation setup

**Fig. 2:**
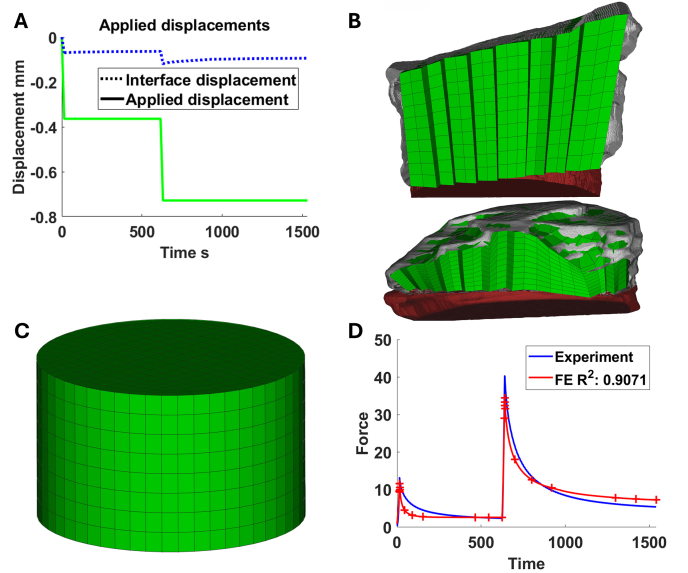
A) Example applied displacement for tomography model based on registration in blue. For comparison the applied load in solid green line. B) Example simplified hexahedral mesh in green, segmented bone (red) and cartilage (gray) included as reference for a healthy (OARSI = 1, upper) and a degenerated (OARSI = 4.5, lower) sample. C) Idealized cylindrical model of the healthy sample in B. D) Example of an ongoing parameter identification. 18 points were used in the RMSE target function, which are marked with +.

The indenter was modelled as an impermeable rigid plate with frictionless contact with the sample. The subchondral bone plate was also assumed to be rigid and impermeable. The outer surface of the sample was assumed to have free fluid flow. To account for the non-linear compliance of the trabecular bone structure in the subchondral bone, a vertical displacement was specified for the rigid subchondral bone plate. The displacement was based on image registration of the motion of the trabecular bone structure from the dynamic images acquired during loading (Fig. 1, Fig. 2A). The movement was applied as a displacement boundary condition. No image was acquired between preloading and the first loading ramp (Fig. 1B); therefore, sample settling during preload cannot be separated from deformation caused by the first loading ramp in this data set. Instead, the movement of the bone-cartilage interface during the first loading ramp was assumed to be 20% of the applied vertical displacement of the indenter. This assumption was based on the registration of another set of cartilage samples, which were also tested in compression. For those, additional images were acquired between preloading and the first loading ramp. This allowed quantifying the bone-cartilage interface displacement during the first loading ramp. The mean interface displacement was 20% (see Supplementary Material for details). The data set contained 12 bone-cartilage units at different stages of OA, according to OARSI gradings, from females tested in a single compression step. The compression caused by the preloading was accounted for by noting the position of the indenter relative to the sample during loading. The compression occurred mainly in the trabecular bone structure.

### 4.3 Mesh and segmentation – tomography model

A mixed mesh with hexahedral and pentahedral poroelastic elements (C3D8P and C3D6P) was created (ANSA v24.1.2, BETA CAE) based on a semi-automatic segmentation of cartilage from the 3D tomographic static images (3D Slicer 5.2.2 [40] and Seg3d 2.5.1 [41]). Smaller features, such as fibrillated tissue were manually removed and smoothed. The top surface of the segmentation was meshed with primarily quadrilateral elements (size = 0.2 mm) and triangular elements where needed to ensure boundary recovery. The surface mesh was then extruded in 10 steps along the cranio-caudal direction (Fig. 2B) into solid elements. This meshing approach was chosen due to the reduced element count and computational cost compared to automatic tetrahedral meshing. Results for mesh convergence and mesh simplification compared with geometrically more detailed tetrahedral meshes are available in Supplementary Fig. S7–9.

### 4.4 Idealized cylinder model – simulation setup

The idealized models were modelled as cylinders (⌀ = 4 mm, sample-specific height) with hexahedral poroelastic elements (C3D8P, Fig. 2C). The base of the model was assumed to be fixed and axial compression was applied based on sample height. All other modelling choices were kept from the tomography model.

### 4.5 Parameter identification

The mechanical parameters were identified by minimizing the normalized root mean square error between the experimental and FE reaction forces for the stress relaxation (Fig. 2D). The minimization problem was solved with a Globalized Nelder-Mead method [42], which uses multiple Nelder-Mead [43] searches to fully cover the parameter space and identify the global minima. Each new Nelder-Meach search was initiated with a starting point that is dissimilar to previous searches based on a probability function [42]. The parameter space was normalized to improve convergence [44]. During the parameter identification, $\nu_{nf}$ was kept constant at 0.42 [45,46] while $E_{nf}$, $E_{0}$*,* $E_{e}$*, M* and *k*_*0*_ were allowed to change. The parameter space and Globalized Nelder-Mead method settings are available in Supplementary Tables 1 and 2.

### 4.6 Statistical analysis

The identified mechanical parameters were analyzed with a linear regression model and the OARSI grade as independent variable. The quality of the fit was investigated with Q-Q and homoscedasticity plots. Log-linear regression was used when the relationship between OARSI grade and the mechanical parameters was non-linear. Homoscedasticity and Q-Q plots are available in Supplementary Fig. S10.

## 5 Results

**Fig. 3:**
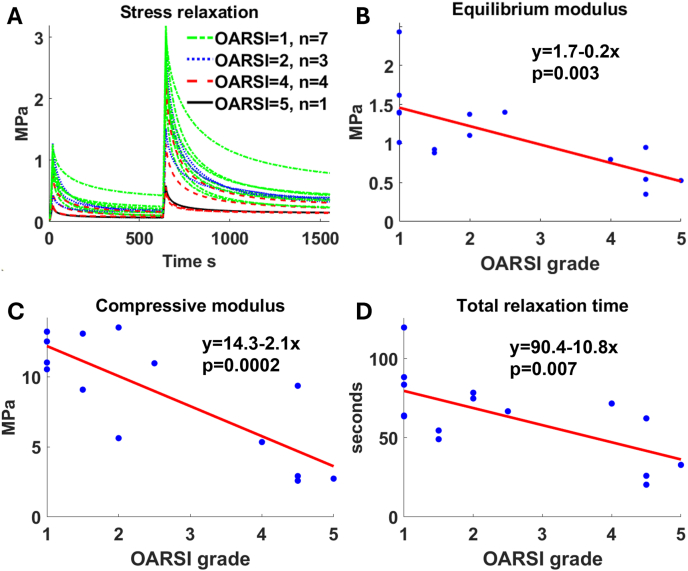
. Analysis of experimental data, A) Stress relaxation for samples used in study, grouped by OARSI grading without subgrading. B–C) Equilibrium E_eq_ and compressive modulus E_i_, D) Total relaxation time T_rel_ for the two stress relaxations.

The result of the experimental data from the stress relaxation test can be seen in Fig. 3A. The stress response between the different sample groups overlapped, especially between the more normal (OARSI = 1) and mildly degenerated (OARSI = 2) groups. Both the compressive and equilibrium modulus decreased with an increasing OARSI grade (Fig. 3B–C), and samples with higher OARSI grade showed a faster relaxation (Fig. 3D).

**Fig. 4:**
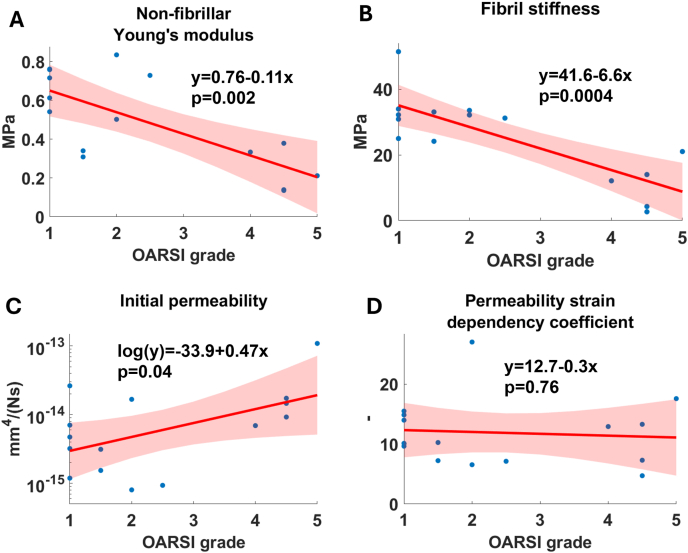
Result of the fitted FRPE material parameters with tomography-based FE models, A) Non-fibrillar Young's modulus E_nf_, B) collagen fibril spring stiffness E_0_, C) logarithmic initial permeability k_0_, D) Strain dependent permeability coefficient M. Confidence intervals (95%) are presented in shaded area.

**Fig. 5:**
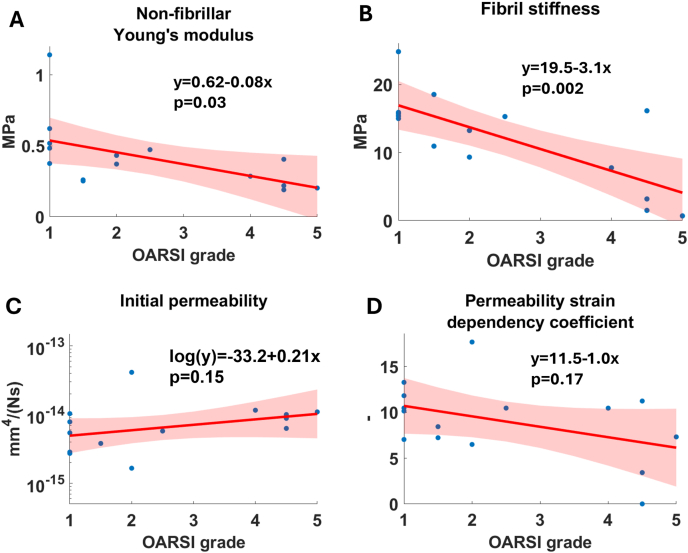
Result of the fitted FRPE material parameters with idealized cylinder models, A) Non-fibrillar Young's modulus E_nf_, B) collagen fibril spring stiffness E_0_, C) logarithmic initial permeability k_0_, D) Permeability strain dependent coefficient M. Confidence intervals (95%) are presented in shaded area.

**Fig. 6:**
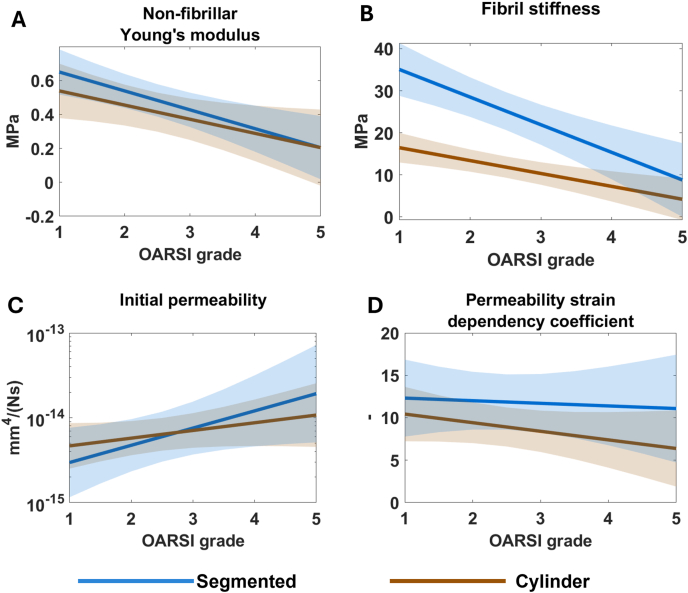
Comparison of the identified parameters (solid lines) for the segmented models and the idealized cylinder models 95% confidence intervals for the predictor function (shaded area). A) Non-fibrillar Young's modulus *E*_*nf*_, B) collagen fibril spring stiffness *E*_*0*_, C) logarithmic initial permeability *k*_*0*_, D) Permeability strain dependent coefficient *M*.

The stress versus time response of the x-ray tomography-based FE models correlated well with the experimental stress-relaxation data (R^2^ = 0.96 ± 0.02, mean ± SD). Both the non-fibrillar Young's modulus *E*_*nf*_ and the fibrillar stiffness *E*_*0*_ showed a decrease in stiffness with an increased OARSI grade (Fig. 4A–B). Initial permeability *k*_*0*_ showed a log-normal distribution and increasing permeability with increased OARSI grade (Fig. 4C). Finally, there was no clear association between the strain dependent permeability *M* and OARSI grade (Fig. 4D). The stress versus time response of the idealized cylinder FE models correlated well with the experimental stress-relaxation data (R^2^ = 0.96 ± 0.02, mean ± SD). Only the non-fibrillar Young's modulus *E*_*nf*_ and the fibrillar stiffness *E*_*0*_ were associated with the OARSI grading and showed a decrease in stiffness with increased OARSI grade (Fig. 5A–B). Initial permeability *k*_*0*_ and strain dependent permeability M showed no clear association with OARSI grade (Fig. 5C–D). A comparison between the predicted parameters for the tomography-based models (Fig. 2) and the idealized cylinder is available in Fig. 6. The predicted properties from the segmented models are compared with identified properties for normal and osteoarthritic human femoral cartilage tested with indentation by Ebrahimi et al. [21] in Table 1.

**Table 1:** Comparison between our fitted parameters and the average identified parameters of Ebrahimi et al., 2021 at OARSI grade 1 and 4 and how much they change with increasing OARSI grade.

	*Study*	*E*_*nf*_ (MPa)	*E*_*0*_ (MPa)	*E*_*fe*_ (MPa)	*k*_*0*_ (10^−15^ m^4^/N)	*M* (−)
Normal OARSI = 0–1 (n = 17)	Ebrahimi et al., 2021	0.45	1.05	4.98	3.29	9.46
Degenerated OARSI = 4 (n = 3)	Ebrahimi et al., 2021	0.04	0.00001	10.06	15.43	3.25
Change OARSI 1→4	Ebrahimi et al., 2021	−11.3x	−105000x	+2.2x	+4.7x	−2.9x
Normal OARSI = 1	Current study	0.65	35.1	–	2.90	12.3
Degenerated OARSI = 4	Current study	0.32	15.4	–	12.1	11.1
Change OARSI 1→4	Current study	−2.1x	−3.1x	–	+5.5x	–

## 6 Discussion

The goal of this study was to characterize the mechanical properties of human femoral cartilage using a fibril-reinforced poroelastic material model with FE models based on SR-PhC-μCT images. We identified the material properties using FE simulation and an optimization algorithm. Material properties were also determined using idealized FE models representing the intended cylindrical geometry prepared during sample preparation.

The SR-PhC-μCT based FE models showed reduced stiffness and increased permeability with higher OARSI score, the idealized FE model showed only a decrease in stiffness.

Both the fitted non-fibrillar Young's modulus *E*_*nf*_ and the equilibrium modulus *E*_*eq*_ (experimental) decreased by ∼50–70% (Fig. 3, Fig. 4, Fig. 5A) when comparing close to normal cartilage (OARSI = 1) to severely degenerated cartilage (OARSI = 5). During equilibrium, compressive loads are mainly carried by the proteoglycans [47], which are known to be depleted with increasing OARSI grade. This aligns with previous findings which observed that the nonfibrillar Young's modulus *E*_*nf*_ and equilibrium modulus *E*_*eq*_ decrease with increased degree of OA [6,17,45]. The collagen stiffness decreased with increased degradation (Figs. 4B and ure 5B). Since all FE models were assumed to have the same tissue composition and structure as healthy cartilage [21], the decrease in stiffness could be a reflection of the collagen network disruption during OA, with changes in both orientation and collagen density [6,9,11,12,14,15]. With the progression of OA, the loss of proteoglycans and collagen results in increased swelling, water content and permeability [6,13,16]. We observed an exponentially increasing initial permeability (Fig. 4C) and faster relaxation times (Fig. 3C) with increasing OARSI grade. Interstitial fluid bears most of the load during rapid loading [48]. With disrupted collagen network and increased permeability, fluid pressurization becomes impaired with increased OARSI grade as can be seen in the decrease of the compressive modulus *E*_*inst*_ (Fig. 3C) and quicker relaxation time (Fig. 3D) due to higher permeability. The strain-dependent permeability exponent *M* showed no association with tissue degradation (Fig. 4D). Previous studies using the FRPE material model also found no association between *M* and OARSI grade in tibial cartilage [45]. However, a statistically significant difference was reported between samples with OARSI grade 2–3 and 4 in patellar cartilage [49]. This could be due to the patella's different properties and mechanical role leading to different OA progression in the tissue [19]. Also, *M* and *k*_*0*_ together have been found to be insensitive to balanced changes in nonlinear biphasic models [50,51], resulting in large confidence intervals for the identified parameters.

The geometry of the cartilage samples affected the identified mechanical properties when comparing sample-specific segmented models with idealized cylindrical models (Fig. 6). Both types of models were equally good at reproducing stress relaxation (mean R^2^ = 0.96). Comparing the results of the parameter identification between the segmented and idealized cylinder FE models; The non-fibrillar matrix modulus is similar for both modelling approaches (Fig. 4, Fig. 5, Fig. 6A). The idealized models assume a fixed bone plate and a flat top surface. These assumptions result in more effective compression of the sample [25] compared to the sample-specific segmented model. Interestingly, both the fibril stiffness for close to normal (OARSI = 1) cartilage and the change (slope) with increased OARSI grade are ∼2x higher in the tomography-based models compared to the idealized models (Fig. 4, Fig. 5, Fig. 6B). The fibrils in the superficial zone are perpendicular to the loading direction and become stretched immediately when the sample is compressed and expand in the cylindrical model, increasing their effectiveness compared to the segmented models, aiding the collagen's role in maintaining high fluid pressure during fast loading [52]. The difference in fluid pressure can be seen in Supplementary Fig. S11. In contrast to the segmented models, the idealized cylindrical models showed no association between the initial permeability and the OARSI grade (Fig. 5). This could be due to the idealized models not accounting for the variability caused by sample geometry [25] where the idealized models do not capture the heterogenous strain field caused by the actual sample geometry and the fluid expulsion resulting from uneven compression. Accounting for sample shape could be more important for degenerated samples since their shape is often more irregular, see Fig. 1, Fig. 2B.

Human femoral cartilage of varying degenerative status (OARSI grade 0 to 4) has previously been investigated computationally with a FRPE material model by Ebrahimi et al., 2021 using indentation testing and idealized FE models. Compared to Ebrahimi, we found a 44% higher non-fibrillar modulus (0.65 vs 0.45 MPa) for OARSI 0–1 and a smaller stiffness reduction at grade 4 (−2.1 × vs −11.3 × ), likely due to geometry influence [25] and differences in preload affecting fibrillated samples. Additionally, the differences in collagen-related parameters for both normal and degenerated samples(Table 1) can be attributed to how unconfined and indentation load the collagen network in the used material model [26] and to the two different spring models used. In the FRPE model [26], stiff primary fibrils are arranged in an arcade pattern alongside isotropically dispersed secondary fibrils. During indentation, loading is concentrated on the superficial primary fibrils. In contrast, the softer secondary fibrils are more engaged under compressive loading, where they constrain lateral expansion, showing the importance of matching load case with what is evaluated in the model. This highlights the importance of aligning the applied load with the evaluated properties in the model, as well as assessing the potential effects of new load cases such as shear. The indentation study uses two springs to represent collagen fibrils, one linear for initial stiffness (*E*_*0*_) and one strain dependent (*E*_*fe*_). The fibrils contribution becomes strain dependent with the non-linear spring. Ebrahimi et al., reported the average initial permeability for OARSI grades 0–1 and 4 (3.29 and 15.43∗10^−15^ m^4^/N respectively) to be within the span of our identified parameters (Fig. 4C–Table 1). They also found no significant change in the strain-dependent permeability coefficient *M* between OARSI grades 0–1 and 4, consistent with our finding of no association.

### 6.1 Parameter identification

For the identification of material parameters, a unique solution of the fitting procedure is required. With complex material models, this is challenging as multiple local minima typically exist in the objective function. Therefore, we adopted a global optimization algorithm [42] using a simplex [43] with 20 unique initial guesses. Similar methods have been shown able to find the global minimum when a sufficient number of initial guesses are used [53,54]. We verified the identification by rerunning the fitting procedure multiple times. Additionally, the complexity of the parameter identification increases with the number of unknown parameters. Therefore, we adopted a simplified material model that does not account for the viscous behavior of the collagen fibrils [26,55,56], to reduce the number of parameters while still capturing the mechanical behavior and ensuring a good fit.

### 6.2 Future usage

Our sample-specific FE models contribute helpful knowledge for how the mechanical properties of femoral cartilage change during compressive loading with increased degeneration of the tissue. Future studies could combine in-situ tomography data with complementary experimental techniques, such as small-angle x-ray scattering [57] to obtain sample-specific collagen network orientation and distribution, and Fourier-transform infrared imaging spectroscopy to determine sample-specific proteoglycan content [38]. This combined approach could help reduce other sources of variability. The identified material parameters can be used in predictive knee joint models with degenerative tissue properties where previously bovine cartilage properties have been used [[58], [59], [60]]. The FRPE parameters for healthy cartilage can be used as a reference when creating and testing artificial cartilage for required mechanical properties.

### 6.3 Limitations

Our main limitation is a relatively low number of individual samples, and that the histological OARSI grade distribution was uneven, e.g., only one donor's sample had grade = 5. For determining mechanical properties having more (∼30–60+) [18,22,45] samples is desired. However, all our samples are from unique donors, increasing the statistical power. The availability of synchrotron beamtime and the complexity of in situ loading highly limits how many samples (∼10–20) can be used.

Secondly, in our statistical model, the OARSI grades were treated as continuous [22] instead of ordinal during the regression due to low sample size. The OARSI grade definition has no connection to mechanical properties and our estimated associations for the mechanical properties are linear while the visible degradation in the OARSI grading scheme [27] is not. Last, a linear model that accounts for changes in mechanical properties can be more easily implemented into predictive FE modelling pipelines [58,59]. No consideration was taken for age dependency of the mechanical properties. Cartilage damage and degeneration can sometimes be very local, resulting in large tissue variability within one donor. Thus, using osteochondral samples for histology adjacent to the tissue tested in-situ added uncertainty. However, both samples were obtained within one 16 mm donor osteochondral plug. Still, a few samples showed large variability, and we excluded two samples that showed clear differences in degradation between the tissues used for histology and mechanical testing (Supplementary Fig. S3).

The initial displacement of the subchondral bone plate is based on registration from other samples tested in compression. Consequently, we mimicked only the vertical displacement of the bone to replicate the displacements while also reducing the amount of uncertainty introduced by this assumption. This data set originated from female donors, who may have slightly lower bone mineral density compared to the men in the main study. However, we did not find any correlation between donor age and deflection. Thus, we believe that deflection was mostly related to sample preparation and affected both sample cohorts equally. As seen in Supplementary Fig. S5, subchondral bone compliance largely varies, which will result in some samples either under- or overestimated stress relaxation forces. This inaccuracy will affect the parameter identification, potentially adding variability to the results.

The material models were fitted in compression. It is therefore unclear how well the identified properties would replicate for other loading conditions. For cartilage, swelling is an important property of the tissue. In this study, the swelling pressure can be assumed to be included in the non-fibrillar modulus *E*_*nf*_. Visually, the difference between intact and degenerated samples was clear in both histology and synchrotron images (Fig. 1D). However, the exact changes in tissue structure and composition for the degenerated samples are unknown. To decouple the material parameters from the tissue composition, which is affected by OA grade [6], all samples were assumed to have the same healthy tissue structure [21].

## 7 Conclusion

We identified the material parameters for a FRPE material based on cartilage composition for human femoral cartilage for both intact and degenerated tissue with FE models based on in-situ mechanical compression with synchrotron tomography images and compared those with traditional models based on a simplistic idealized cylindrical shape. In addition, we determined mechanical properties directly from the mechanical data. Regression analyses of the mechanical properties confirmed a reduction in stiffness, increased permeability and faster relaxation with increasing degree of tissue degradation. The identified parameters and their alteration with increasing OARSI grade differed between idealized and segmented models, showing the importance of accurately accounting for the sample shape when identifying mechanical properties. Future studies could use in-situ imaging to better represent experimental conditions.

## CRediT

***Writing – original draft:*** VJ. ***Writing – review & editing:*** LG, AG, MP, HD, CS, AS, ME, HI. ***Visualization:*** VJ. ***Numerical methodology:*** VJ, LG, AG, HI. ***Numerical investigation:*** VJ. ***Experimental investigation:*** MP, HD, AS, CS, ME, HI. ***Conceptualization:*** VJ, LG, AG, HI. ***Supervision:*** LG, AG, HI. ***Data curation:*** MP, HD, AS. ***Resources:*** CS. ***Provision/selection of samples:*** ME. ***Histological investigation:*** AS. ***Project administration and funding acquisition:*** HI.

## Generative AI

During the preparation of this work the author(s) used Copilot in order to check spelling and grammar. After using this tool/service, the author(s) reviewed and edited the content as needed and take(s) full responsibility for the content of the publication.

## Conflict of interest

The authors declare no conflict of interest.
