# Supplementary material for: Degeneration dependent changes in human knee cartilage mechanical properties revealed by synchrotron tomography based finite element modeling
Source: Osteoarthr Cartil Open. 2026 May 27;8(3):100825. doi: 10.1016/j.ocarto.2026.100825 (PMC13262150; doi:10.1016/j.ocarto.2026.100825)
Supplement: Multimedia component 1 [file mmc1.pdf]

Supplementary material

DEGENERATION DEPENDENT CHANGES IN HUMAN KNEE CARTILAGE MECHANICAL PROPERTIES REVEALED BY SYNCHROTRON TOMOGRAPHY BASED FINITE ELEMENT MODELING

Viktor Jönsson[1], Lorenzo Grassi[1], Anna Gustafsson[1], Maria Pierantoni[1], Hector Dejea[1,2], Amanda Sjögren[3], Christian M. Schlepütz[4], Martin Englund[3], Hanna Isaksson[1]

1. Department of Biomedical Engineering, Lund University, Lund, Sweden
2. MAX IV Laboratory, Lund University, Lund, Sweden
3. Clinical Sciences Lund Orthopedics, Lund University, Lund, Sweden
4. Swiss Light Source, Paul Scherrer Institute, Villigen, Switzerland

https://doi.org/10.1016/j.ocarto.2026.100825

Compressive modulus

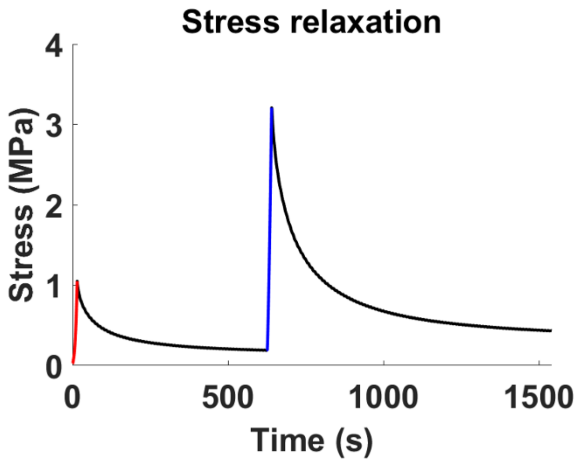

Supplementary figure S1. *Based on the stress–strain data recorded during the loading ramps (red and blue), the compressive modulus is determined as the slope (green) of the stress–strain curve. The two ramps were combined to have continuous data.*

Sample preparation

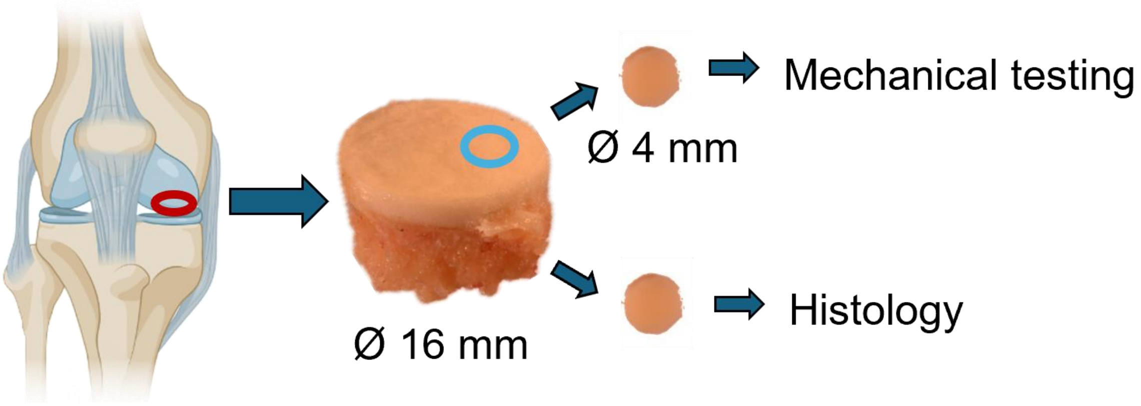

Supplementary figure S2. *Example plug (⌀ = 16 mm, red circle) extracted from the load-bearing area of the femoral medial condyle. From this plug, a ⌀ = 4 mm core (blue circle) was taken to the synchrotron for in situ testing, and another part was used for histology.*

Sample exclusion

Images of the two excluded samples with large differences in degeneration between histology and synchrotron images are in supplementary figure S2. Examples of samples excluded due to cracks in the sample are in supplementary figure S3. The sample excluded for overloading the load cell had OARSI grade 0.

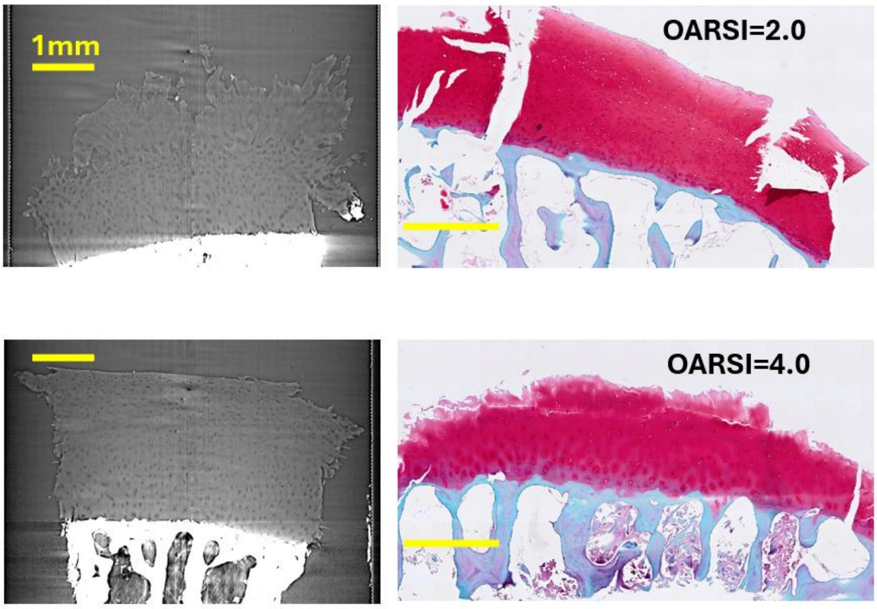

Supplementary figure S3. *Two samples were excluded because of large differences in the extent of degeneration observed in the histology and synchrotron images. Scalebars are 1 mm.*

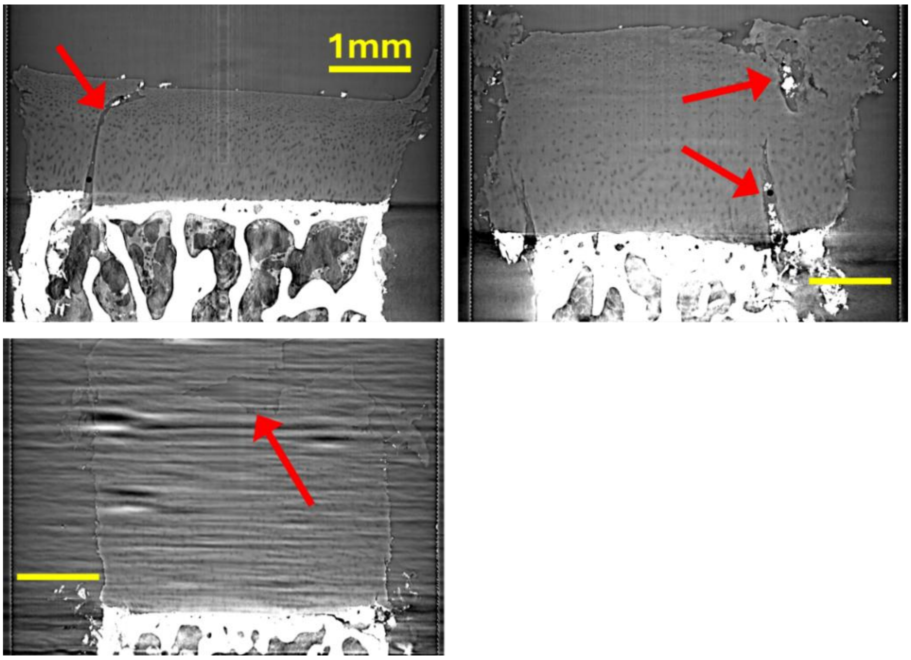

Supplementary figure S4. *Samples excluded due to damage, assumed to originate from sample preparation. Arrows point at damaged parts of samples. Scale bar represents 1mm.*

Boundary condition

The results of registering the second data set of experimental data are in Supplementary figure S4.The second data set used osteochondral plugs (N=12, ⌀=4mm) tested in compression using the same synchrotron-based in-situ imaging approach, but with the addition of one extra image set being acquired right after preloading, before the first loading ramp. Since the applied displacement is dependent on the sample's height, the displacement of the bone plate has been normalized.

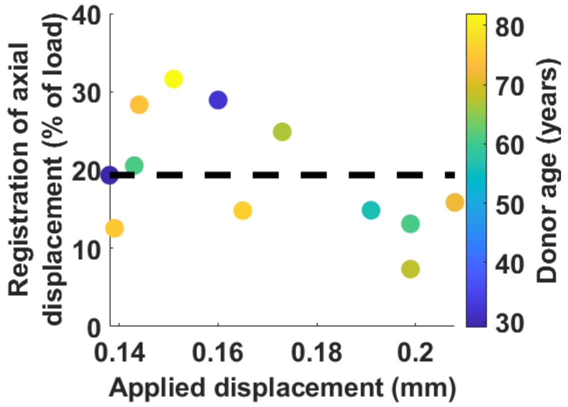

Supplementary figure S5. *Displacement of the bone-cartilage interface during first loading ramp from the 2nd data set with mechanical testing of articular cartilage. No trend was found between the donors or the applied load to the amount of movement of the bone-cartilage interface. Instead, the interface was assumed to move with 20% of the applied displacement.*

This assumption was necessary as modelling the bone as compliant only for the 2nd ramp yielded disproportionately large forces for the first ramp compared to the second ramp. The highly non-linear behavior and sensitivity to changes in effective applied load for the material model can be seen in supplementary figure S5.

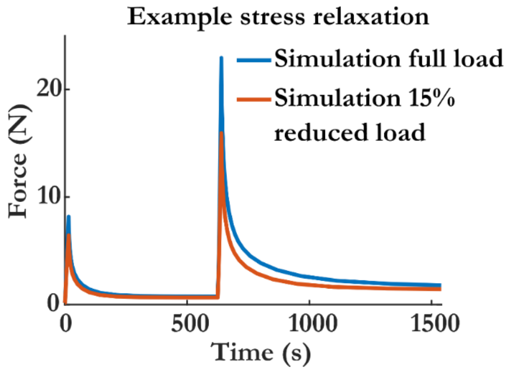

Supplementary figure S6. *Example of stress relaxation sensitivity when the applied load is effectively reduced because of the compliance of the subchondral bone, evaluated using FE simulation. Here the subchondral bone plate moves downward axially with 15% of the applied load. The equilibrium and peak forces are reduced by ~20-40%.*

Mesh simplification

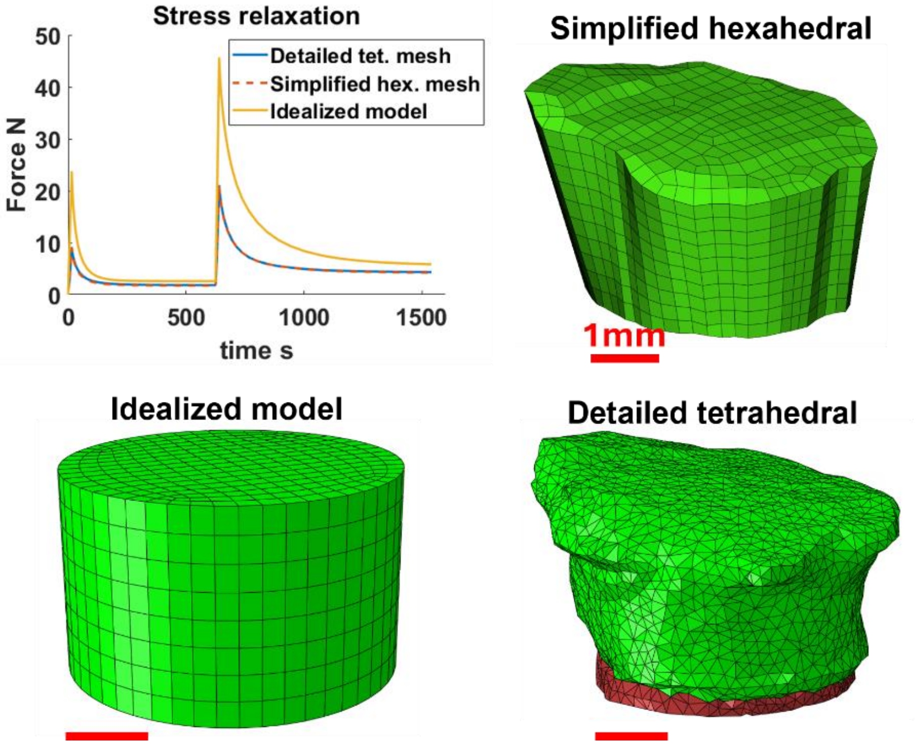

Supplementary figure S7. *Result of mesh simplification for a sample with OARSI grade 1 with regular geometry. Comparison between detailed tetrahedral mesh (subchondral bone visualized in red), simplified hexahedral mesh and idealized model. The idealized model assumes rigid subchondral bone during loading. The peak and equilibrium force differences between detailed tetrahedral and simplified hexahedral are <5%. The tetrahedral model has 41540 elements, whereas the simplified hexahedral has 2981 elements. Scale bars are 1 mm.*

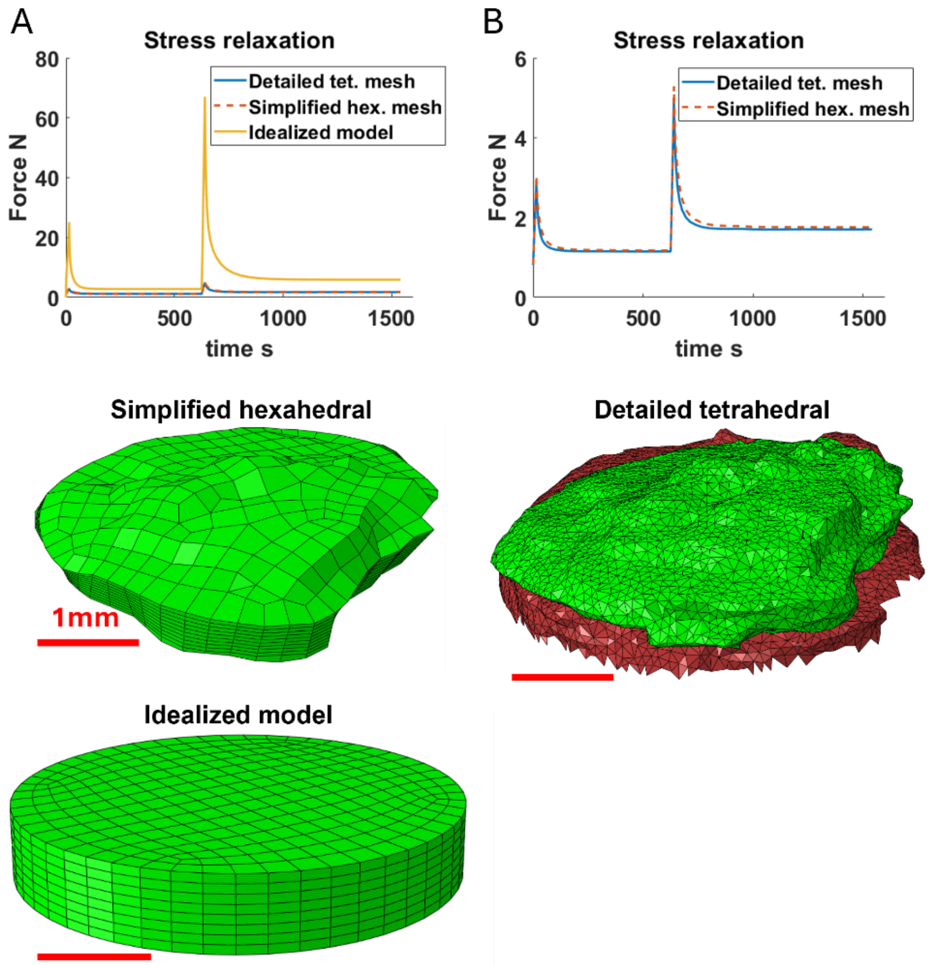

Supplementary figure S8. *Result of mesh simplification for sample with severe OA (OARSI grade 5) and complex geometry. Comparison between detailed tetrahedral mesh (subchondral bone visualized in red), simplified hexahedral mesh and idealized model in plot A. The idealized model assumes rigid subchondral bone during loading. The detailed and simplified models are compared in plot B. Peak force difference between detailed tetrahedral and simplified hexahedral is <8%, whereas the equilibrium forces differ <5%. The simplified hexahedral model has 2450 elements, whereas the tetrahedral model has 48725 elements. Scale bars are 1 mm.*

Mesh convergence

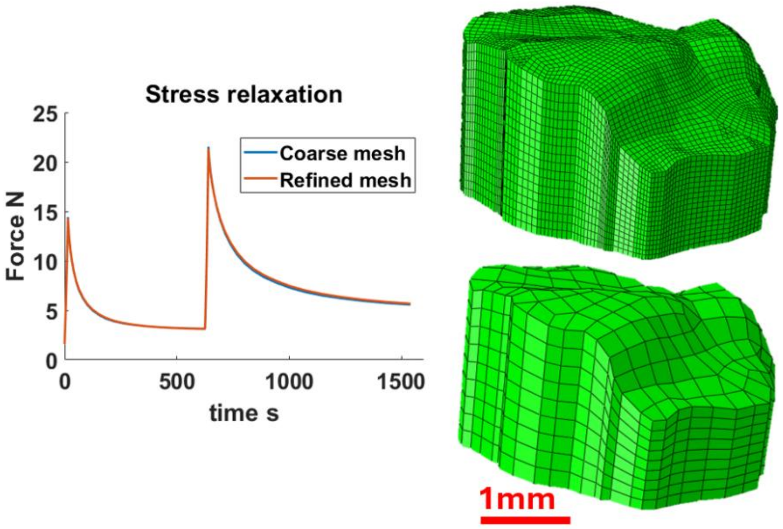

Supplementary figure S9. *Example mesh convergence for sample, Coarse mesh has 2420 elements, refined has 45940. Difference in peak and equilibrium forces <5%. Scale bar is 1 mm.*

Parameter identification settings

Settings used for the optimization algorithm (Globalized Nelder-Mead method) and parameter limits.

Setting name	***value***
maxRestarts	*20*
maxEvals	*5000*
nPoints	*8*
maxIter	*150*
alpha	*1*
beta	*0.4*
gamma	*3*
epsilon	*5E-6*
Sigma	*5E-3*

Supplementary table S1. *Used Globalized Nelder-Mead settings.*

Parameter	*Enf (MPa)*	*E0 (MPa)*	*K0* ($mm^4/(Ns)$)	*M (-)*
Minimum	*0.01*	*0.01*	*0.0001*	*0.1*
Maximum	*1.5*	*60*	*0.40*	*30*

Supplementary table S2. *Lower and upper limit for parameters during fitting.*

Linear regression and diagnostic plots

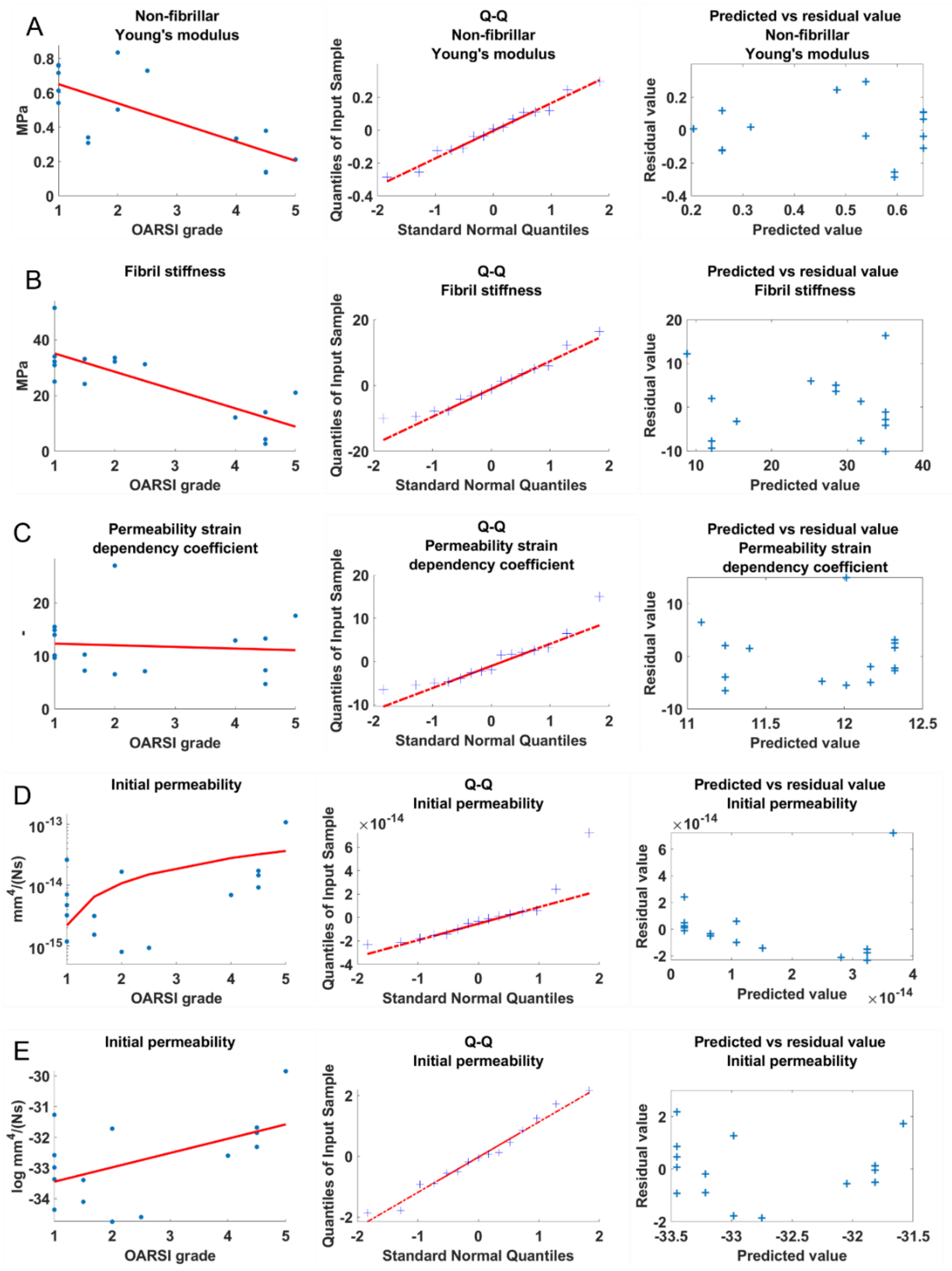

Supplementary figure 10*: Left column, identified parameters with estimated association, middle column Q-Q plot and homoscedasticity plots in right column for A) Non-fibrillar Young's modulus E_{nf} B) Fibril stiffness E_0 C) Permeability stain dependency coefficient M, D) Initial permeability k_0 with linear regression (D) and log-linear regression(E). Based on the Q-Q and homoscedasticity plots for the linear regression of initial permeability (supplementary figure 8D,E) the log-linear regression was chosen due to its more normally distributed residuals.*

Supplementary results

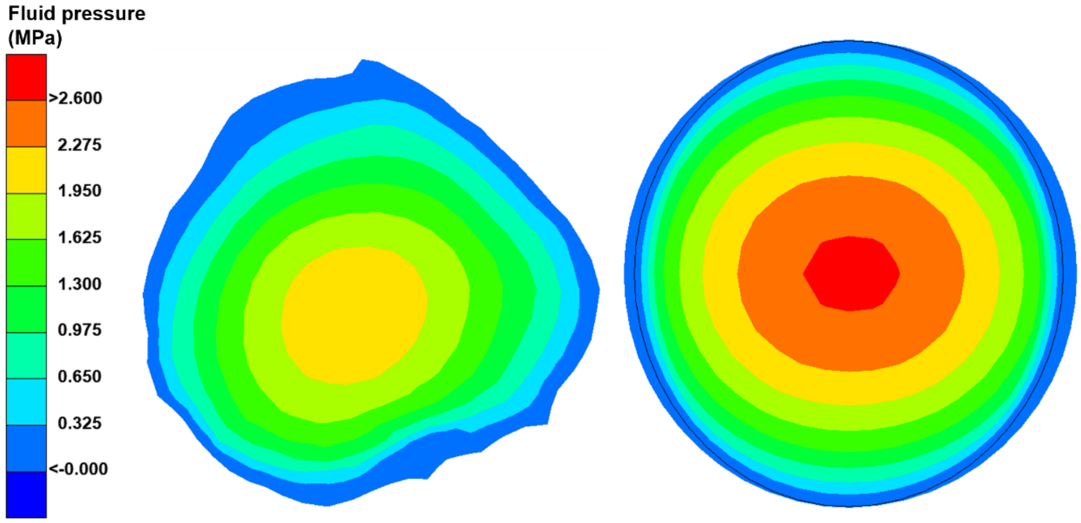

Supplementary figure 11. *Fluid pressure seen on top of samples (left segmented sample, right ideal sample) at end of the first loading ramp for a normal (OARSI=1) sample. Both models share the same material parameters. If each model had its own set of parameters (from optimization), the difference in pressure would be smaller*.
